# A Comparative Assessment of Different Aerogel-Insulated Building Walls for Enhanced Thermal Insulation Performance

**DOI:** 10.3390/gels9120943

**Published:** 2023-11-30

**Authors:** Jianming Yang, Huijun Wu, Yuying Liang, Jian Cen, Xianyong Zhang

**Affiliations:** 1 School of Automation, Guangdong Polytechnic Normal University, Guangzhou 510665, Chinazhangfriendjun@163.com (X.Z.); 2 School of Civil Engineering, Guangzhou University, Guangzhou 510006, China; 3 Department of Architecture and Civil Engineering, City University of Hong Kong, Hong Kong, China; 4 Intelligent Building Equipment Information Integration and Control Key Laboratory, Guangzhou 510665, China

**Keywords:** aerogel, building, energy-efficient, thermal insulation, wall

## Abstract

Aerogel is widely recognized as a superinsulating material with great potential for enhancing the thermal insulation performance of building walls. It can be applied in various forms such as aerogel plasters (AP), aerogel fibrous composites (AFC), and aerogel concrete (AC) in practical engineering applications. This study aims to investigate the most efficient application form for maximizing building insulation performance while minimizing the amount of aerogel used. To predict the thermal insulation performance of aerogel-insulated walls, a resistance–capacitance network model integrating the aerogels’ effective thermal conductivity model was developed and was validated by comparing it with Fluent simulation software results in terms of surface temperature. Using the validated models, the thermophysical parameters, transient thermal properties, and transmission load were predicted and compared among AP, AFC, and AC walls. The results indicate that using AFC can result in approximately 50% cost savings to achieve the same thermal resistance. After adding a 20 mm thickness of aerogel to the reference wall without aerogel, the AFC wall exhibited the highest improvement in thermal insulation performance, reaching 46.0–53.5%, followed by the AP wall, and then the AC wall, aligning with considerations of microstructural perspectives, thermal resistance distributions, and thermal non-uniformity factors. Therefore, giving priority to AFC use could reduce the required amount of silica aerogel and enhance economic efficiency. These results provide valuable insights for theoretical models and the application of aerogel-insulated walls in building engineering insulation.

## 1. Introduction

Thermal insulation plays a crucial role in improving building energy efficiency and reducing costs associated with heating, ventilation, and air conditioning [1,2]. In recent decades, there has been a growing interest in advanced materials that offer exceptional insulation performance [3,4]. Among these materials, silica aerogel has emerged as a highly promising option due to its outstanding thermal insulation properties [5,6]. This unique aerogel material, prepared using the sol–gel method, possesses an open-cell network structure consisting of interconnected particles and air pockets [7,8]. Renowned for its remarkably low density and low thermal conductivity, aerogel is an ideal candidate for enhancing the thermal insulation performance of building walls [9,10].

A wide range of aerogel-insulated products have been developed and implemented in practical building engineering [11]. These encompass aerogel-insulated plasters (AP) [12], aerogel fibrous composites (AFC) [13], and aerogel-insulated concrete (AC) [14]. Based on the functional structure of a building exterior wall, the aerogel application forms can be classified into three categories: AP as surface coatings, AFC as insulation layers, and AC as thermal heavyweight layers. Extensive studies have been conducted on the preparation, characterization, and applications of these three types of aerogel-insulated walls in building structures [15].

The AP combines aerogel particles with a binder, offering a flexible and easy-to-apply solution for wall insulation [16]. It primarily consists of gel solutions or aerogel particles applied to building surfaces using spraying, brushing, or other methods. He et al. [12] prepared an aerogel-based coating and found that a 4 mm thick coating could significantly reduce the surface temperature from 351 °C to only 188 °C, demonstrating a significant thermal insulation improvement. Ibrahim et al. [17] studied a 5 cm aerogel-based plaster on the exterior surface of a building test cell under real weather conditions. They found that the aerogel-based plaster showed better performance than other insulating materials due to higher thermal resistance and capacitance. They further evaluated the effect of the plaster thickness through experimental and numerical measures [18] and determined that the optimum thickness is 1.7–4.4 cm for payback period of 1.4–2.7 years.

The AFC incorporates cylindrical fibers within an aerogel matrix, with each fiber well wrapped in aerogel [19], resulting in enhanced mechanical strength and insulation performance [20]. Zhou and Hsieh successfully engineered cellulose-rich fibers to create novel cellulose nanofibril aerogel [21]. It exhibits high specific tensile strength to act effectively as a thermal insulator across a wide range of working temperatures (−20 °C to 150 °C) for energy-saving building structures. Joly et al. [22] prepared AFC by combining silica aerogel and needled glass fibers, resulting in superinsulation with a thermal conductivity of 0.016 W·m^−1^·K^−1^ and excellent compressive stress of 38.6 MPa. Yang et al. [23] numerically and experimentally investigated the thermal performance of AFC in building energy efficiency by utilizing AFC as insulation layers for exterior walls. The results suggested that the AFC wall exhibited a ~20% decrease in the fluctuation amplitude of the internal temperature compared with traditional insulating walls.

The AC integrates aerogel particles into the concrete mixture, providing insulation properties to the structural material itself [24]. Fickler and co-authors developed a high-performance aerogel concrete by controlling the amount of silica aerogel and the storage conditions [25]. They found that with 60 vol% aerogel, the AC exhibited a density of 860 kg·m^−3^, compressive strength of 10 MPa, and a thermal conductivity of 0.170 W·m^−1^·K^−1^. Liu et al. [26] produced a novel foam concrete reinforced with silica aerogel using the sol–gel technique and vacuum technologies. The aerogel could fill up to 74 vol%, evenly distributed throughout the porous structure of the foam concrete. The composite demonstrated a 48.4% lower thermal conductivity while maintaining a compression strength of 1.12 MPa. Zhang et al. [27] prepared aerogel-foamed concrete and compared its thermal performance to ordinary concrete and expandable polystyrene. They concluded that the novel aerogel concrete reduced thermal losses by approximately one-third and exhibited better thermal insulating performance than traditional ones.

While these different forms of aerogel applications have demonstrated potential in enhancing thermal insulation, it is essential to identify the most efficient application form that maximizes insulation performance while minimizing the quantity of aerogel required. This consideration becomes particularly important from an economic standpoint, as aerogel is a relatively expensive material and its extensive use in building insulation significantly raises application costs [28,29].

This study thus investigated and compared the thermal insulation performance of different forms of aerogel insulation in building walls. Our aim was to determine the application form that offers the highest insulation efficiency while minimizing the amount of aerogel used. To achieve this, we developed and validated a resistance–capacitance model that integrates an effective thermal conductivity model for aerogels. By comparing the thermal insulation performance of different aerogel-insulated walls, this study will contribute to the development of more cost-effective and efficient insulation solutions, promoting energy conservation and sustainability in buildings.

## 2. Results and Discussion

### 2.1. Predicting Effective Thermal Conductivity and Thermal Resistance of Aerogel-Insulated Structures

**Figure 1:**
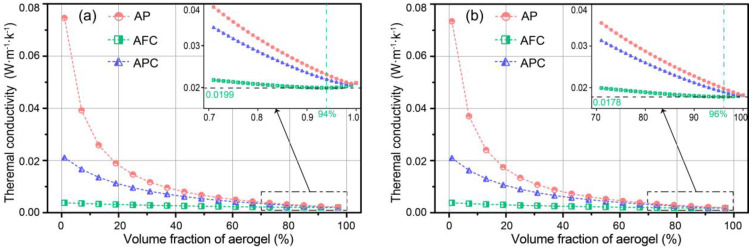
Effective thermal conductivity of aerogel-insulated walls for various volume fractions at aerogel density of (**a**) 100 kg·m^−3^, (**b**) 150 kg·m^−3^.

Figure 1a,b depict the variations in effective thermal conductivity of different types of aerogel-insulated structures (i.e., AP, AFC, and AC) with varying volume fractions at aerogel densities of 100 and 150 kg·m^−3^, respectively. The thermal conductivities of AP and AC decrease rapidly with increasing volume fraction of aerogel, but the rate of decrease diminishes gradually, as observed in Figure 1a. In contrast, the thermal conductivity of AFC decreases slowly, and within the range of 80% to 99% volume fraction, it first increases and then decreases with a minimum of 0.0199 W·m^−1^·K^−1^ at volume fraction of 94%. This is attributed to the presence of fibers, which reduces the radiative thermal conductivity of the aerogel.

In comparison, at an aerogel density of 150 kg·m^−3^, the thermal conductivity of aerogel decreases from 0.021 to 0.018 W·m^−1^·K^−1^. This decrease is due to the higher density, which reduces the extinction coefficient and radiative heat transfer coefficient of the aerogel, resulting in a lower thermal conductivity. Furthermore, the decrease is also due to the effect of the pore size, which changes with the aerogel density and volume fraction [30,31]. As presented by Merillas et al. [32], there exists an optimum particle size lower than 40 nm for obtaining effective insulating PUR-PIR aerogels (λ < 20 mW·m^−1^·K^−1^). The thermal conductivity of AFC shows a minimum of 0.0178 W·m^−1^·K^−1^ at volume fraction of 96%, which is lower than those of AP and AC with minimums of 0.0185 and 0.0183 W·m^−1^·K^−1^, respectively. Additionally, the effective thermal conductivity of aerogel-insulated structures with greater aerogel density of 150 kg·m^−3^ is lower than those with 100 kg·m^−3^.

**Figure 2:**
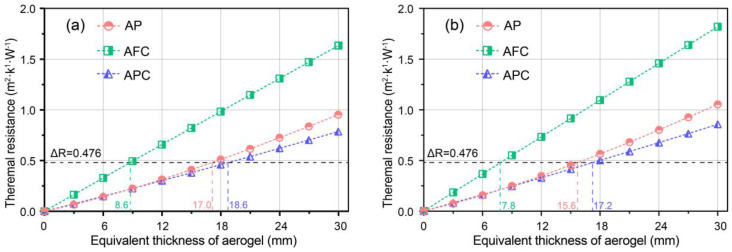
Incremental thermal resistance of aerogel-insulated walls for various equivalent thicknesses of aerogel at aerogel density of (**a**) 100 kg·m^−3^, (**b**) 150 kg·m^−3^.

Furthermore, the variation of incremental thermal resistance with equivalent thickness of aerogel was determined for three aerogel-insulated walls, as shown in Figure 2a,b, with densities of 100 and 150 kg·m^−3^, respectively. From Figure 2a, it can be observed that as the equivalent thickness increases, the thermal resistance increment exhibits a linear growth pattern. When the thickness increases from 0 to 30 mm, the thermal resistance increments for AP, AFC, and AC walls reach 0.954, 1.635, and 0.787 m^2^·K·W^−1^, respectively. Thus, the order of increasing thermal resistance increments is AC, AP, and AFC. With an increase in aerogel density, the thermal resistance increments for the three different forms of aerogel-insulated walls also increase, reaching 1.053, 1.819, and 0.855 m^2^·K·W^−1^, respectively, corresponding to improvements of 10.4%, 11.3%, and 8.6%.

According to the energy-saving design standards for public buildings, the required thermal resistance increment for exterior walls in cold regions is 0.476 m^2^·K·W^−1^, based on the traditional GF-insulated (i.e., glassfiber porous insulation) wall as a reference. When incorporating different forms of aerogel materials, the required equivalent thicknesses of aerogel are 17.0, 8.6, and 18.6 mm, respectively. This means that the AFC wall requires approximately 50.6% less aerogel consumption compared with the AP wall, and approximately 53.8% less compared with the AC wall. Similarly, when using a density of 150 kg·m^−3^, the required equivalent thicknesses of aerogel are 15.6, 7.8, and 17.2 mm, respectively, with the AFC wall reducing aerogel consumption by approximately 50.0% and 53.7%. When the preparation and maintenance costs for the three forms of aerogel are comparable, adopting the AFC structure can save approximately 50% of the material usage compared with the other two structures.

**Figure 3:**
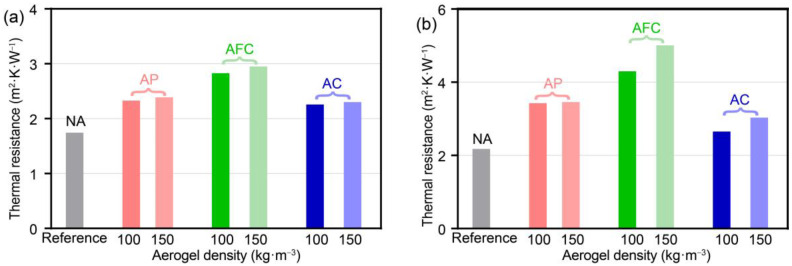
Thermal resistance of aerogel-insulated walls at aerogel density of 100 and 150 kg·m^−3^. (**a**) Steady-state thermal resistance; (**b**) transient equivalent thermal resistance.

To further compare the thermal impeding capacity of different forms of aerogel-insulated walls, the same equivalent thickness of 20 mm of aerogel was used, which satisfies the energy-saving design standards. The walls’ thermal resistances in steady state are compared in Figure 3a. The AFC wall exhibits the highest steady-state thermal resistance, with values of 2.829 and 2.952 m^2^·K·W^−1^ for aerogel densities of 100 and 150 kg·m^−3^, respectively. These values are significantly higher than the reference wall’s thermal resistance of 1.746 m^2^·K·W^−1^ and higher than the thermal resistances of aerogel coating and aerogel–concrete walls with the same thickness.

In contrast, the transient equivalent thermal resistance better reflects the insulation ability of the exterior wall under actual outdoor meteorological conditions. The transient equivalent thermal resistance ($R_{eq}$, [m^2^·K·W^−1^]) through aerogel-insulated walls during a calculation period Tp can be calculated by [33]:

(1) $$R_{eq}=\frac{\int_{0}^{Tp}\left|T_{ws,ex}-T_{ws,in}\right|d\tau }{\int_{0}^{Tp}q_{ws,in}d\tau }$$

The calculation results are shown in Figure 3b. It can be observed that the equivalent thermal resistance values of different forms of aerogel-insulated walls in dynamic environments are higher than the steady-state thermal resistance. The AFC wall exhibits the highest equivalent thermal resistance, reaching 4.299–5.010 m^2^·K·W^−1^, and it also shows the largest increase compared with the steady-state thermal resistance. On the other hand, the increase in thermal resistance is smaller for the AC wall, with values of 2.650 and 3.033 m^2^·K·W^−1^, respectively, and even smaller for the AP wall, with values of 3.426 and 3.458 m^2^·K·W^−1^. Therefore, it can be suggested that the AFC wall offers the best capacity in thermal impeding for both steady-state and dynamic environments.

### 2.2. Modelling Transient Thermal Performance of Aerogel-Insulated Walls

**Figure 4:**
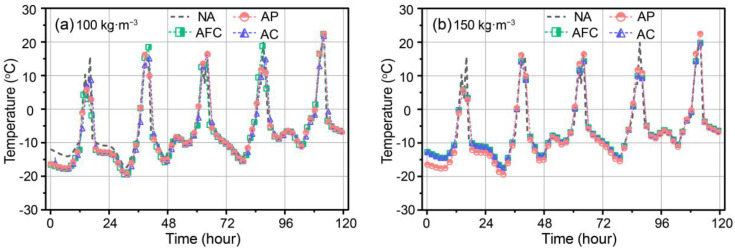
Transient exterior surface temperature of aerogel-insulated walls at aerogel density of (**a**) 100 kg·m^−3^ and (**b**) 150 kg·m^−3^.

The transient temperatures of the exterior surfaces of the AP, AFC, and AC walls were modelled and compared with the reference GF-insulated wall (non-aerogel, referred to as NA), with aerogel densities of 100 and 150 kg·m^−3^, as shown in Figure 4a,b, respectively. Throughout the modeling process, a consistent thickness of 20 mm equivalent aerogel was used for the AP, AFC, and AC walls. The wall structure with the thermophysical parameters described in Table 1 was used, whose thermal conductivity and specific heat were measured using the transient hot-strip (THS) method with a Hot Disk TPS2500 instrument from Sweden. The measurements were conducted at room temperature of 25 °C and atmospheric pressure. The combined standard deviation of the THS technique was found to be less than 3% [23]. The initial values of interior air temperature and wall temperature were set to 18 °C for all cases. The external environment boundary was set to Beijing, specifically considering five typical cold days (18–22 January) selected from the Thermal Environment Analysis database of Chinese Meteorological Data. These setups were also used for subsequent modelling.

Observing Figure 4a, the transient exterior surface temperature exhibited periodic fluctuations ranging from −19.7 to 23.4 °C over five periods. The maximum values occurred around 14 to 17 p.m., while the minimum values occurred around 5 to 8 a.m. These fluctuations result from solar radiation during the daytime and radiative cooling from the night sky. Similar variations were observed for different aerogel-insulated walls and were comparable to the overall outdoor air temperature. Notably, on the first day, the exterior surface temperature of the reference wall was higher than that of the aerogel-insulated walls, indicating better temperature impedance from the aerogel-insulated walls. For example, the minimum at 7.2 a.m. (−14.1 °C) and the maximum value at 16.2 p.m. (15.6 °C) for the NA wall were greater than those of the aerogel-insulated walls, suggesting lower heat flux through the exterior surface of the aerogel-insulated walls.

In comparison, as shown in Figure 4b for an aerogel density of 150 kg·m^−3^, the general variations in the transient exterior surface temperature are consistent with those at 100 kg·m^−3^ density. However, except for the AP wall, the AC and AFC walls exhibited fluctuations similar in magnitude to the NA wall. This indicates reduced fluctuation amplitude for the aerogel-insulated concrete wall. As a result, the AP wall showed a smaller fluctuation amplitude compared with the other walls throughout the calculation period. However, the timing of the peak and minimum values remained consistent, corresponding to the outdoor climate parameters. This suggests that varying aerogel density and type does not affect the timing of peaks and minimums.

**Figure 5:**
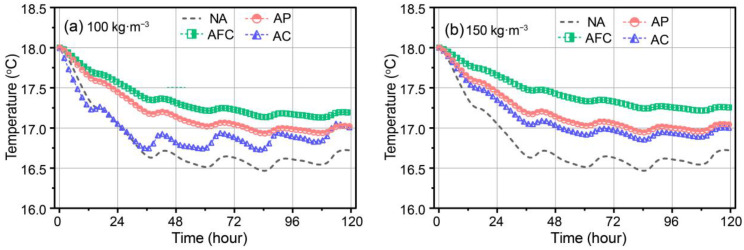
Transient interior surface temperature of aerogel-insulated walls at aerogel density of (**a**) 100 kg·m^−3^ and (**b**) 150 kg·m^−3^.

The transient temperatures of the interior surfaces of the AP, AFC, and AC walls were modelled for aerogel densities of 100 and 150 kg·m^−3^, as shown in Figure 5a,b, respectively. At 100 kg·m^−3^ density in Figure 5a, the temperature variations of the reference and AC wall were quite consistent over the first 40 h, rapidly decreasing from 18 °C to approximately 16.8 °C. The interior surface temperature of the AFC wall gradually decreased from 18℃ to 17.3 °C, while the AP wall decreased to 17.2 °C. Subsequently, periodic temperature fluctuations occurred, with the AFC wall having the smallest amplitude, followed by the AP wall. Although the AC and reference walls exhibited larger fluctuations, the fluctuation center was greater for the AC wall at 16.6 °C versus 16.8 °C for the reference wall, but still lower than those of the AP and AFC walls at 17.0 °C and 17.2 °C, respectively.

In contrast, at 150 kg·m^−3^ density in Figure 5b, the overall trend remained unchanged, while the AC wall fluctuation amplitude reduced significantly, approaching that of the AP wall. Over the first 40 h, the temperature decreased from 18 °C to 17.0 °C, and then fluctuated around 16.9 °C. This indicates improved dynamic thermal insulation performance of the AC wall with increased aerogel density. Similarly, the AP fluctuation amplitude decreased, but the AFC wall fluctuation center increased noticeably to 17.5 °C. This suggests AFC exhibits the best performance for maintaining stable indoor temperature fluctuations, followed by AP, and then AC.

**Figure 6:**
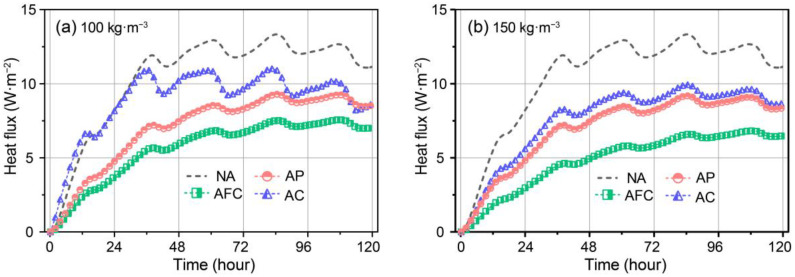
Transient heat flux of the interior surfaces of aerogel-insulated walls at aerogel density of (**a**) 100 kg·m^−3^ and (**b**) 150 kg·m^−3^.

The transient heat flux values of the interior surfaces of the AP, AFC, and AC walls were modelled for aerogel densities of 100 and 150 kg·m^−3^, as shown in Figure 6a,b, respectively. In Figure 6a, at 100 kg·m^−3^, the heat flux initially increased rapidly and then fluctuated around a central value. For instance, during the first 40 h, the heat flux values of the NA and AC walls both experienced rapid increases, reaching approximately 14.1 and 12.4 W·m^−2^, respectively from an initial value of 0. In comparison, the heat flux of the AFC wall gradually increased to 5.5 W·m^−2^, while the AP wall increased to 7.2 W·m^−2^. Subsequently, periodic heat flux fluctuations occurred, with the AFC wall having the smallest amplitude and center at 7.1 W·m^−2^. The AP wall exhibited slightly greater fluctuations around a center of 8.8 W·m^−2^. Although both the AC and reference walls exhibited larger fluctuations, their centers differed noticeably at 10.0 and 12.4 W·m^−2^, respectively.

As the aerogel density increased to 150 kg·m^−3^, although the overall trend remained unchanged, the AC wall’s fluctuation amplitude reduced significantly and approached that of the AP wall. During the first 40 h, the heat flux increased to 7.8 W·m^−2^ and then fluctuated slightly more than that of the AP wall. Similarly, the increased aerogel density resulted in a noticeable decrease in the temperature fluctuation center of the AFC wall, reaching approximately 6.0 W·m^−2^. This suggests improved dynamic thermal insulation in the AC and AFC walls with increased aerogel density. Overall, AFC demonstrates the best transient thermal performance, followed by AP and then AC.

### 2.3. Comparing Thermal Insulation Index of Different Aerogel-Insulated Walls

To assess the time difference between reaching peak temperatures at the exterior and interior surfaces of an aerogel-insulated wall [34,35], the time lag based on the extremum of exterior and interior surface temperatures was calculated as:

(2) $$\begin{array}{c} {∆\tau }_{l}=\tau_{i,ma}-\tau_{e,ma} \end{array}$$

where $\tau_{i,ma}$ and $\tau_{e,ma}$ [h] are the times when the interior and exterior surface temperatures reach their maximum values, respectively.

**Figure 7:**
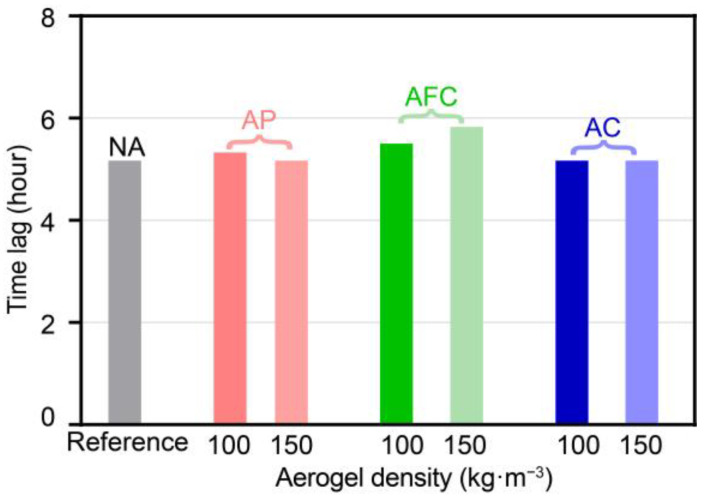
Comparisons of time lag of aerogel-insulated walls.

Figure 7 illustrates the time lag comparisons for aerogel-insulated walls at aerogel densities of 100 and 150 kg·m^−3^. The time lag of the NA wall is 5.2 h, which aligns with that of the AC wall at 100 kg·m^−3^ density, as well as the AP and AC walls at 150 kg·m^−3^ density. In contrast, the AFC wall at 150 kg·m^−3^ density exhibits the maximum time lag of 5.8 h, surpassing the AC wall at 100 kg·m^−3^ density with 5.3 h, and the AFC wall at 100 kg·m^−3^ density with 5.5 h. This suggests that aerogel density increase does not absolutely impact the time lag of aerogel-insulated walls. Generally, the aerogel structure slightly delayed the time lag compared with the NA wall.

Furthermore, to evaluate the attenuation extent of temperature waves, the decrement factor of the aerogel-insulated walls was calculated by comparing the amplitude of temperature fluctuation on the interior and exterior surfaces, with the following equation:

(3) $$\begin{array}{c} F_{d}=\frac{T_{i,ma}-T_{i,mi}}{T_{e,ma}-T_{e,mi}}\times 1000‰ \end{array}$$

where $T_{i,ma}$ and $T_{i,mi}$ [°C] are the maximum and minimum temperatures on the interior surface, respectively, $T_{e,ma}$ and $T_{e,mi}$ [°C] are the maximum and minimum temperatures on the exterior surface, respectively.

**Figure 8:**
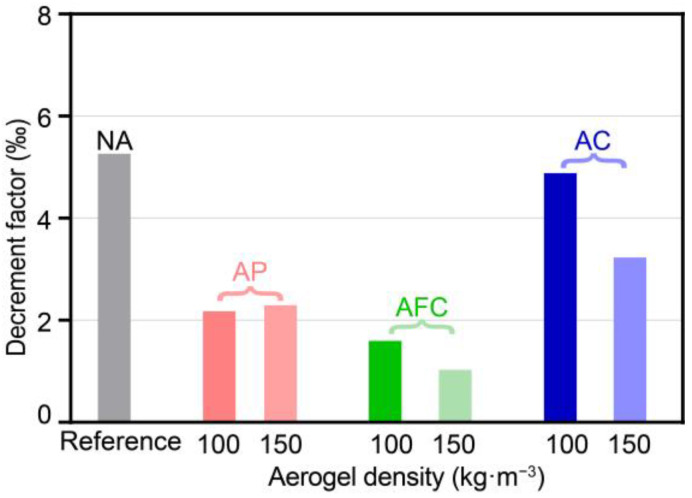
Comparisons of decrement factor of aerogel-insulated walls.

Figure 8 illustrates the comparisons of decrement factor for aerogel-insulated walls at the aerogel densities of 100 and 150 kg·m^−3^. The decrement factors of the aerogel-insulated walls fall within the range of 1.03‰ to 4.88‰, all of which are smaller than the NA wall’s decrement factor of 5.27‰. This indicates that the insulated aerogel structures have a greater capacity to mitigate temperature fluctuations. The AFC wall exhibits the lowest decrement factors of 1.03‰ and 1.60‰ at 100 and 150 kg·m^−3^, respectively. The AP wall follows with decrement factors of 2.17‰ and 2.29‰, while the AC wall has decrement factors of 4.88‰ and 3.23‰. To decrease the decrement factor, it is advisable to decrease the aerogel density for the AFC and AP walls while increasing density for the AC.

**Figure 9:**
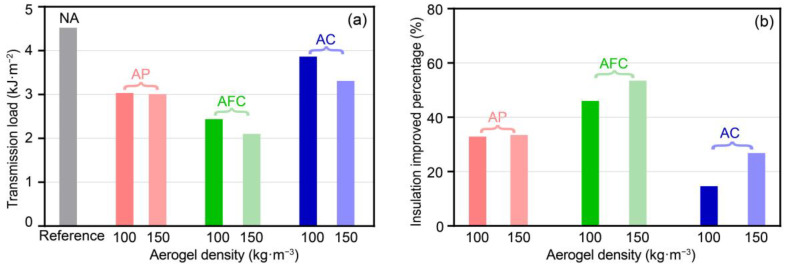
Comparisons of transmission load through aerogel-insulated walls and insulation improvement percentage compared with NA wall. (**a**) Transmission load and (**b**) insulation improved percentage.

The transmission load across aerogel-insulated walls and their comparison to the NA wall are presented based on the aforementioned heat flux and Equation (12), as depicted in Figure 9a, for aerogel densities of 100 and 150 kg·m^−3^. The AFC wall demonstrates the lowest transmission load, with values of 2.44 and 2.10 kJ·m^−2^ for aerogel densities of 100 and 150 kg·m^−3^, respectively. Following that, the AP wall exhibits transmission load of 3.03 and 3.01 kJ·m^−2^, while the AC wall shows transmission load of 3.86 and 3.31 kJ·m^−2^. These findings suggest that increasing the aerogel density is advisable for achieving improved thermal insulation performance. Notably, the transmission loads of aerogel-insulated walls ranging from 2.10 to 3.86 kJ·m^−2^ are lower than that of the NA wall (4.52 kJ·m^−2^). This indicates that insulated aerogel structures significantly reduce the transmission load. Overall, the addition of a 20 mm aerogel layer results in a greater reduction in transmission load for the AFC wall, followed by the AP wall, while the AC wall exhibits the least improvement in thermal insulation performance.

To further quantitatively evaluate the percentage reduction ($P_{i}$) in transmission load of aerogel-insulated walls compared with the reference NA wall, the calculation expression is:

(4) $$\begin{array}{c} P_{i}=\frac{Q_{aN}-Q_{ai}}{Q_{aN}}\times 100\% \end{array}$$

where subscript i represents different forms of aerogel walls, $Q_{N}$ [kJ·m^−2^] is the NA wall’s transmission load.

As depicted in Figure 9b, the addition of a 20 mm aerogel layer in various forms leads to improved thermal insulation performance compared with the NA wall, specifically, 32.9~33.5% for the AP wall, 46.0~53.5% for the AFC wall, and 14.6~26.9% for the AC wall. Moreover, increasing the density from 100 to 150 kg·m^−3^ improves the thermal insulation performance by 0.6%, 7.5%, and 12.3% for the AC, AFC, and AP walls, respectively. This indicates that increasing the aerogel density has a more significant impact on the thermal insulation of the AC and AFC walls, while its effect on the AP wall is limited. We attribute this observation to the thermal nonuniformity factor. The AP wall has a smaller thickness of exterior plaster layer compared with the concrete layer and insulation wall. As a result, the AP wall exhibits a higher nonuniformity factor compared with the AC and AFC walls. When we increase the aerogel density, although the thermal resistance increments of different aerogel-insulated walls are comparable (Figure 3a), it further increases the nonuniformity factor of the aerogel walls. According to our results, an increase in the nonuniformity factor is associated with a greater impact on thermal performance, such as the transmission load, in walls with a lower nonuniformity factor. However, walls with a higher nonuniformity factor have limited potential for further improvement in thermal insulation performance [33]. Therefore, increasing the aerogel density has a more significant impact on the thermal insulation provided by AC and AFC walls compared with an AP wall. Additionally, considering the same aerogel production process and preparation cost, AFC structures should be prioritized to minimize aerogel usage and enhance thermal insulation efficiency, while reducing the use of AC walls.

**Figure 10:**
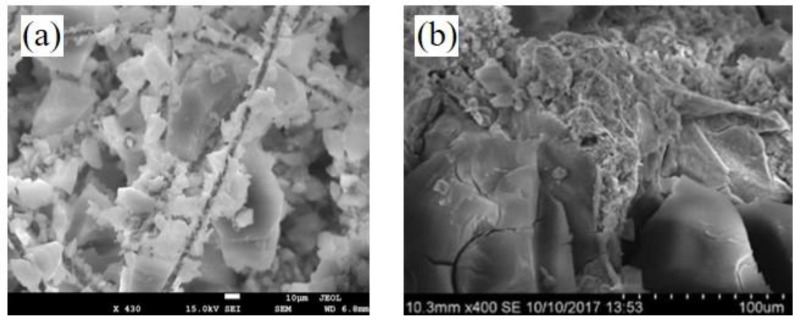
Scanning electron micrographs of aerogels in forms of (**a**) AFC structure and (**b**) AC (or AP) structure [37].

Based on the aforementioned comparisons, it is evident that adding aerogel materials in different forms results in varying levels of improved thermal insulation performance. The AFC structure demonstrates the highest performance, followed by the AP structure, and finally the AC structure. This difference in performance can be attributed to its unique microstructural integration. In the AFC structure, the aerogel serves as the matrix, and each fiber is effectively enveloped and dispersed within the aerogel matrix, as illustrated in reference [36]. Since the fibers are long and oriented perpendicular to the direction of heat transfer, there are no thermal bridging pathways [30,32], as illustrated in the SEM image in Figure 10a. In contrast, the AP and AC walls have a plaster and concrete matrix, respectively, with the aerogel added in a particulate form and dispersed. In these struc-tures, thermal bridging pathways still exist [31], as shown in Figure 10b [37]. Therefore, from a microstructural perspective, the AFC structure clearly outperforms the AP and AC structures and exhibits superior thermal insulation efficiency.

When comparing the AP and AC structures, the AP structure was superior to the AC structure. One major reason for this may be the distribution of thermal resistance. Extensive studies have shown that placing thermal insulation materials on the exterior of walls is more effective than placing them on the interior [38]. Since aerogel is a high-thermal-resistance material, placing it on the exterior plaster layer provides better thermal insulation performance than placing it in the internal concrete layer. Additionally, the second major reason may be the thermal nonuniformity factor. Wu et al. [33] discovered that engineering walls with a high nonuniformity factor can significantly reduce the wall heat flux. As the thickness of the exterior plaster layer is much smaller than that of the porous concrete layer, adding the same amount of aerogel has a greater impact on the nonuniformity factor of the plaster layer, resulting in a higher thermal resistance nonuniformity factor for the AP structure compared to the AC structure. Therefore, considering both the distribution of thermal resistance and the thermal nonuniformity factor, it is reasonable to conclude that the AP structure outperforms the AC structure and exhibits superior thermal insulation efficiency.

## 3. Conclusions

In this study, the application forms of aerogel in walls were categorized into AP, AFC, and AC, whose thermal insulation performance was predicted and compared, with the following important conclusions drawn: First, effective thermal conductivity models of aerogel materials in various forms and dynamic thermal resistance–capacitance models were developed and were validated through theoretical comparisons and Fluent simulations. Second, the effects of aerogel content and density on thermal conductivity and wall thermal resistance increment were predicted. The AFC structure demonstrated the highest thermal resistance and offered potential cost savings compared with the AP and AC structures. Third, the addition of a 20 mm aerogel layer in different forms improved surface temperature stability and heat flux for the AP and AC walls, with the AFC wall showing the most significant enhancements. Finally, the AFC structure outperformed the AP and AC structures in terms of thermal insulation efficiency, as evidenced by its highest thermal resistance and superior surface temperature stability. Considering identical aerogel production and cost, prioritizing AFC structures can minimize aerogel usage while maximizing thermal insulation efficiency.

In future work, it is important to consider the integration of different factors such as mechanical properties, manufacturing processes, construction and transportation costs, waterproofing, and sound insulation, in order to optimize the overall performance of aerogel composites for practical applications.

## 4. Methodology

### 4.1. Overview of the Methodology

**Figure 11:**
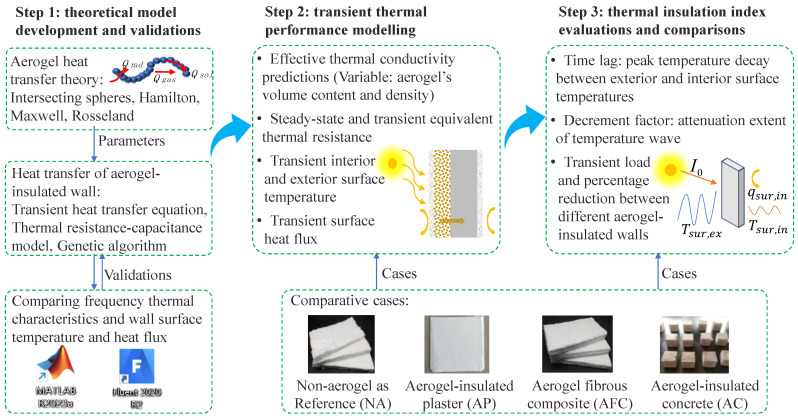
Methodology of the study.

The methodology used to investigate and compare the thermal insulation performance of different aerogel-insulated building walls is depicted in Figure 11. Initially, the heat transfer theory and predictive models for the effective thermal conductivity of aerogel and its insulated structures are presented. The obtained thermal conductivity results are then utilized as input for the thermal resistance–capacitance model, which is developed to simulate the thermal insulation performance of aerogel-insulated building walls. To ensure accuracy and reliability, the developed model is validated against Fluent simulation software by examining frequency thermal characteristics, wall surface temperature, and heat flux.

Using the validated models, we conducted transient thermal performance modelling of the AP, AFC, and AC walls, comparing them with a reference wall without aerogel (NA), which is a traditional glassfiber (GF) blanket-based wall. Predictions were made for the effective thermal conductivity under different aerogel volume contents and densities, steady-state and transient equivalent thermal resistance, and transient thermal properties. Finally, thermal insulation indices were evaluated and compiled, including the time lag (the time it takes for the peak temperature to decay between the exterior and interior surface temperatures) and the decrement factor (the extent of attenuation of the temperature wave), as well as comparisons of transient load and percentage reduction among different aerogel-insulated walls.

### 4.2. Heat Transfer Model of Aerogel-Insulated Structures

**Figure 12:**
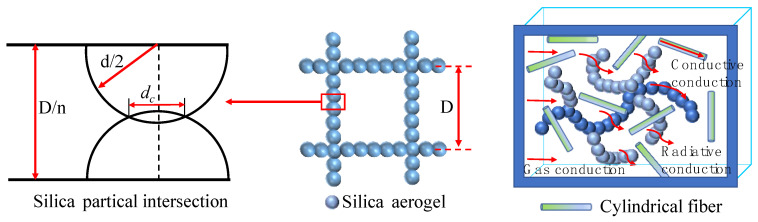
Heat transfer mechanism within silica aerogels.

The aerogel has micro-sized silica particles with diameters typically ranging from 1 to 5 nm, while the interconnected air pores within the aerogel usually have sizes between 5 and 50 nm, which are smaller than the average free path of air molecules under atmospheric pressure (~74 nm). This indicates that gas molecules within the aerogel pores are restricted in their movement compared with free space, as shown in Figure 12. Based on kinetic theory, the thermal conductivity of gas molecules ($\lambda_{c,g}$, [W·m^−1^·K^−1^]) in porous aerogels can be expressed as [39]:

(5) $$\begin{array}{c} \lambda_{g}=\frac{0.461\times 10^{5}\left(2.25c_{p}/c_{v}-1.25\right){\left(8pN_{g}/\pi m_{g}\right)}^{1/2}}{lm}\frac{C_{v}}{N_{A}} \end{array}$$

where subscript g represents gas molecules,$\;c_{p}$ [J·kg^−1^·K^−1^] and $c_{v}$ [J·m^−3^·K^−1^] are the specific heat of constant pressure and constant volume, respectively, p [Pa] is gas pressure, $N_{g}$ and $N_{A}$ are the air molecule number density and molecule number, $m_{g}$ [kg] is the mass of air molecules, lm [m] is the average free path of gas molecules, which can be calculated as [39]:

(6) $$\begin{array}{c} lm=1/0.25S\rho \varphi^{-1}+\sqrt{2}N_{g}\pi d_{g}^{2} \end{array}$$

where S is specific surface area, ρ [g·cm^−3^] is volume density, φ is porosity, and $d_{g}^{\;}$ is the diameter of air molecules.

Within the aerogel there is a coupling effect of thermal conduction between the restricted gas molecules and silica particles. The gas–solid coupled thermal conductivity ($\lambda_{gs}^{\;}$, [W·m^−1^·K^−1^]) of the aerogel can be calculated based on the intersecting sphere model, expressed as follows:

(7) $$\begin{array}{c} \lambda_{gs}^{\;}=\frac{\pi \lambda_{g}^{\;}S}{2n_{s}r_{\lambda }D}\left[-\sqrt{1-r_{d}^{2}}-\frac{D}{n_{s}r_{\lambda }d}\ln(1-\frac{n_{s}r_{\lambda }d}{D}\sqrt{1-r_{d}^{2}}\;)\right]+\frac{d_{c}\lambda_{s}^{\;}}{1.1n_{s}D} \end{array}\;\;\;\;\;\;+\frac{(n_{s}-1)\pi \lambda_{g}^{\;}d}{r_{\lambda }D}(r_{d}^{2}-1+\frac{D}{r_{\lambda }d}\ln\frac{D-r_{\lambda }d_{c}}{D-r_{\lambda }d})+(1-\frac{d}{D}{)}^{2}\lambda_{g}^{\;}$$

where subscripts s and gs represent solid and coupled gas–solid, respectively; d, D, and $d_{c}$ [m] are the particle diameter, pore diameter, and contact diameter between two adjacent silica particles, respectively, $n_{s}$ is the number of spherical particles on each side length, $r_{d}^{\;}$ is the ratio of $d_{c}$ to d, $\lambda_{s}^{\;}$ [W·m^−1^·K^−1^] is the solid thermal conductivity of silica particles, $r_{\lambda }$ is the ratio of $\lambda_{s}^{\;}$ to $\lambda_{g}^{\;}$.

After combining with the plasters, porous fibers, and foam concrete, the overall thermal conductivity of the aerogel-insulated structures (i.e., AP, AFC, and AC) is expressed as the sum of the conduction and radiation thermal conductivities, represented as:

(8) $$\lambda_{o}=\lambda_{c}+\lambda_{r}$$

where the subscripts o, c, and r represent the overall conductivity, conduction, and radiation, respectively. Typically, aerogel-based composites have a significant optical thickness, and the radiation thermal conductivity can be calculated using the Rosseland equation [40,41]:

(9) $$\lambda_{r}=\frac{16n_{T}^{2}\sigma T^{3}}{3\beta_{a}}$$

where $n_{T}$ is the temperature-dependent effective refractive index, σ is the Stefan–Boltzmann constant, T [K] is the ambient temperature, $\beta_{a}$ [m^−1^] is the aerogel extinction coefficient.

Regarding the conductive thermal conductivity of AP and AC structures, as the aerogel particles are uniformly dispersed in a particle form, forming a uniformly distributed isotropic structure, the coupled thermal conductivity can be calculated using the Maxwell model [42]:

(10) $$\lambda_{c}=\left[1+\frac{3\left(\lambda_{gs}^{\;}-\lambda_{c,m}\right)v_{m}}{\left(\lambda_{gs}^{\;}+2\lambda_{c,m}\right)-\left(\lambda_{gs}^{\;}-\lambda_{c,m}\right)v_{m}}\right]\lambda_{gs}^{\;}$$

where $\lambda_{c,m}$ [W·m^−1^·K^−1^] is the thermal conductivity of the matrix, i.e., plasters or foam concrete, $v_{m}$ is the volume fraction of the matrix. For the AP structure in this study, only aerogel particles bonded with plasters to the exterior surface are considered, without the addition of other fillers or additives.

The conductive thermal conductivity of the AFC structure depends on the conductive thermal conductivities of the aerogel and fibers and their volume fractions. Considering the isotropic structure of aerogels and the random distribution of fibers within the aerogel matrix perpendicular to the direction of heat flow, the Hamilton model can be used to predict the conductive thermal conductivity [43]:

(11) $$\begin{array}{c} \lambda_{c}=\frac{\lambda_{c,f}+\left(C_{s}-1\right)\lambda_{gs}^{\;}+\left(C_{s}-1\right)\left(\lambda_{c,f}^{\;}-\lambda_{gs}^{\;}\right)v_{f}}{\lambda_{c,f}+\left(C_{s}-1\right)\lambda_{gs}^{\;}+\left(\lambda_{c,f}-\lambda_{gs}^{\;}\right)v_{f}}\lambda_{gs}^{\;} \end{array}$$

where $v_{f}$ is the volume fraction of the porous fibers, $C_{s}$ is the shape factor (which is 6 for long straight cylindrical fibers), $\lambda_{c,f}$ [W·m^−1^·K^−1^] is the thermal conductivity of the fibers.

### 4.3. Development of Thermal Resistance-Capacitance Model for Insulation Performance of Aerogel-Insulated Walls

The thermal insulation performance of aerogel-insulated walls is primarily evaluated over a time period (Tp, [hour]) through the transmission load (TL, [J·m^−2^]) at the inner surface of the wall. The calculation equation is expressed as [44]:

(12) $$\begin{array}{c} TL=\int_{0}^{Tp}h_{in}\left[T_{ws,in}\left(\tau \right)-T_{in}\left(\tau \right)\right] \end{array}$$

where $h_{in}$ [W·m^−2^·K^−1^] is the convective heat transfer coefficient on the interior surface of the aerogel-insulated wall, which is determined as a function of the temperature difference in accordance with the correlations provided in the *ASHRAE Handbook* [45]. $T_{ws,in}\left(\tau \right)$ and $T_{in}\left(\tau \right)$ [°C] denote the transient temperatures at the wall’s interior surface and the indoor air, respectively. The TL directly reflects the aerogel-insulated wall’s ability to resist external temperatures and heat flow, with a smaller value indicating better dynamic thermal insulation performance.

**Figure 13:**
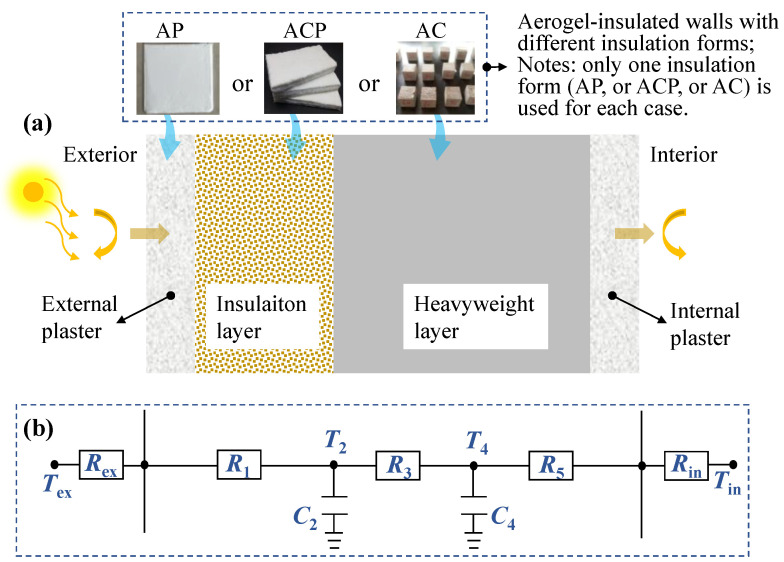
Structure and model of aerogel-insulated wall. (**a**) Insulative structure, (**b**) resistance–capacitance nodal model.

To determine the inner surface’s transient temperature, generally considering that the wall dimensions in the thickness direction are greater than 10 times the wall thickness, a one-dimensional transient heat conduction along the thickness direction is used, which can be described by:

(13) $$\frac{\partial T\left(x,\tau \right)}{\partial \tau }=a_{\;}\frac{\partial^{2}T\left(x,\tau \right)}{\partial x_{\;}^{2}}$$

where a [m^2^·s^−1^] is the thermal diffusivity of wall structure, $T\left(x,\tau \right)$ [°C] is used to record the temperature at time τ [s] and at the position x [m] of the wall. Each wall layer consists of different materials for the AP, AFC, and AC walls is shown in Figure 13a. At any given time τ, the heat flux ($q_{\;}$, [W·m^−2^]) through the position x can be expressed as:

(14) $$q_{\;}\left(x_{\;},\tau \right)=-\lambda_{x}\frac{\partial T\left(x,\tau \right)}{\partial x}$$

where $\lambda_{x}$ [W·m^−1^·K^−1^] is the thermal conductivity of the layer in position x.

A simplified thermal nodal model with three resistances and two capacitances was developed to solve the above transient heat transfer equation. Figure 13b shows the resistance–capacitance distribution schematic, where nodes 2 and 4 record the distribution of resistances and capacitances, with nodal temperatures $T_{2}\left(\tau \right)$ and $T_{4}\left(\tau \right)$ [°C], nodal resistances $R_{1}$, $R_{3}$, and $R_{5}$ [m^2^·K·W⁻^1^], and nodal capacitances $C_{2}$ and $C_{4}$ [kJ·K⁻^1^·m⁻^2^]. From this, the corresponding relationship between heat flow and temperature can be developed with the following node equations [33,46]:

(15) $$\begin{array}{c} C_{2}\frac{dT_{2}}{dt}=\frac{T_{ws,ex}\left(\tau \right)-T_{2}\left(\tau \right)}{R_{1}}-\frac{T_{2}\left(\tau \right)-T_{4}(\tau )}{R_{3}} \end{array}$$

(16) $$\begin{array}{c} C_{4}\frac{dT_{4}}{dt}=\frac{T_{2}\left(\tau \right)-T_{4}(\tau )}{R_{3}}-\frac{T_{4}(\tau )-T_{ws,in}(\tau )}{R_{5}} \end{array}$$

(17) $$q_{ws,ex}=\frac{T_{ws,ex}\left(\tau \right)-T_{2}(\tau )}{R_{1}}$$

(18) $$q_{ws,in}=\frac{T_{4}(\tau )-T_{ws,in}(\tau )}{R_{5}}$$

where subscripts ws, ex, and in represent wall surface, exterior, and interior, respectively; $q_{s,ex}$ and $q_{s,in}$ denote the heat flux of the exterior and interior surfaces of the wall, respectively.

The parameters distribution of $R_{1},R_{3},R_{5}$ and $C_{2},C_{4}$ for the resistance–capacitance model is optimized in the frequency domain, expressed as follows [46]:

(19) $$\begin{array}{c} f\left(R_{1},C_{2},R_{3}\right)=\mathrm{Min}\left[\frac{1}{J\left(R_{1},C_{2},R_{3}\right)}\right] \end{array}$$

where the objective function J is calculated by:

(20) $$\begin{array}{c} J\left(R_{1},C_{2},R_{3}\right)=\sum_{n=1}^{Np}\sum_{m=X,Y,Z}(W_{m}^{AM}\left|\left|G_{m}\left(j\omega_{n}\right)\right|-\left|G_{m}^{'}\left(j\omega_{n}\right)\right|\right|\\ \;\;\;\;\;\;\;\;\;\;\;\;\;\;\;\;+W_{m}^{PL}\left|PL\left|G_{m}\left(j\omega_{n}\right)\right|-PL\left|G_{m}^{'}\left(j\omega_{n}\right)\right|\right|)\; \end{array}$$

where n is the frequency exponent (10^−n^) and Np is the frequency points [47]. PL is phase lag, W is weighting factors, and $G_{m}$ and $G_{m}^{'}$ are functions of frequency characteristics. The symbols m=X,Y,Z represent the matrices for external, cross, and internal heat conduction, respectively [48]. In this study, the genetic algorithm was utilized for the nonlinear optimization problem [49].

## 5. Validation of the Theoretical Method

The aforementioned theoretical approach incorporates predictive models for the effective thermal conductivity of aerogel-insulated materials, genetic algorithms for thermal resistance–capacitance parameter identification, and a resistance–capacitance network model for the thermal performance analysis of aerogel-insulated walls. The thermal conductivity models for various aerogel-insulated materials had been validated against experimental measurements in our previous studies; refer to [50] for AP, reference [13] for AFC, and reference [37] for AC.

The accuracy of the genetic algorithm for thermal resistance–capacitance parameter identification of aerogel-insulated walls was validated by comparison with traditional theoretical models using MATLAB programming. The external thermal disturbances on the wall surface, including outdoor air temperature, solar radiation, and sky radiation, have dominant periodicities ranging from several hours to one year, with corresponding angular frequency magnitudes of 10^−7^−10^−4^ rad·s^−1^. Hence, the consistency between the thermal resistance–capacitance identification method and theoretical reference model was examined in terms of the frequency-domain thermal characteristics (phase angle and amplitude) within this frequency range.

**Figure 14:**
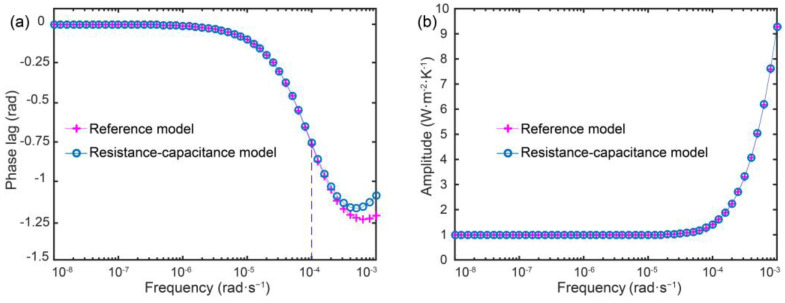
Comparisons of frequency response characteristics of interior surface. (**a**) Phase lag, (**b**) amplitude.

Taking AFC as an example, the phase angle and amplitude of the internal wall surface transfer matrix were compared between the two methods, as depicted in Figure 14a,b, respectively. During the simulations, a typical four-layered building wall commonly used in cold regions of China was taken as an example. The multilayer structure from inside to outside includes the interior plaster (20 mm), foam concrete (200 mm), aerogel insulation, and exterior plaster (20 mm). The aerogel-insulated layer utilizes AFC with a thermal conductivity of 0.0208 W·m^−1^·K^−1^. The other structural materials’ thermophysical properties are listed in Table 1 [37,51].

Figure 14a shows that within the low-frequency region of 10^−8^–10^−6^ rad·s^−1^, the phase angle remains stable at around 0 rad. As the angular frequency increases, the phase angle first decreases then increases, reaching minimums of −1.2 rad and −1.25 rad at around 5 × 10^−3^ rad·s^−1^, as predicted by the thermal network and theoretical reference models, respectively, slightly larger than the internal transfer matrix phase angle at the same frequency. As illustrated in Figure 14b, the amplitude of the internal wall surface transfer matrix remains stable at around 1.0 W·m^−2^·K^−1^ within the angular frequency range of 10^−8^–10^−4^ rad·s^−1^, consistent in magnitude and trend with the exterior wall surface transfer matrix.

Compared with the theoretical reference model, the simplified thermal network model shows some deviation in predicting the internal transfer matrix phase angle around 10^−3^ rad·s^−1^. However, within the angular frequency range of 10^−7^ to 10^−4^ rad·s^−1^, the predicted phase angles from both models are in good agreement. Moreover, the predicted amplitude values of the internal transfer matrix from both models are consistent across the entire computational frequency range. This demonstrates the good reliability of the identified thermal resistance–capacitance parameters in predicting the frequency-domain thermal characteristics of the internal transfer matrix.

Furthermore, the accuracy of the resistance–capacitance model for transient thermal performance of aerogel-insulated walls was validated through dedicated simulations using Fluent software. During the simulation, both the initial interior air temperature and the wall temperature distribution were set to 18 °C. The exterior air temperature boundary conditions were set to a periodic temperature wave with a 24 h period. The temperatures throughout the exterior wall over a continuous period of a week were simulated. The nodal temperatures and heat fluxes predicted by the resistance–capacitance nodal model at the interior and exterior surfaces of the wall were compared against the Fluent simulation results.

**Figure 15:**
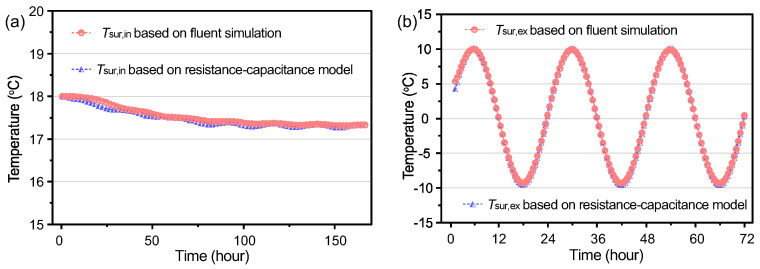
Comparisons of temperature distribution between thermal resistance–capacitance nodal model and Fluent simulations. (**a**) Interior surface temperature, (**b**) exterior surface temperature.

Figure 15a,b show the temperature distribution comparisons between the thermal resistance–capacitance model and Fluent simulations for the exterior surface temperature and interior surface temperature, respectively. The exterior surface nodal temperatures fluctuate periodically according to the periodic external temperature wave boundary, while the variation of the inner surface nodal temperatures is smaller, indicating that the outer fluctuations have less influence on the inner thermal performance, i.e., the dynamic adiabatic performance is superior. The difference in exterior surface temperatures between the resistance–capacitance model and Fluent simulations at peak values is within 0.5 °C. For the interior surface temperature, it remains in the range of 18.0–17.4 °C, with minimal fluctuation. There is minor difference between the early trends of the thermal resistance–capacitance model and Fluent simulations, because of the absence of initial values in the resistance–capacitance model. Overall, the interior surface temperature predicted by the resistance–capacitance model is slightly lower than the Fluent simulation, within 0.5 °C, thus validating the developed thermal resistance–capacitance model for aerogel-insulated walls.

In summary, both the genetic algorithm for thermal resistance–capacitance parameter identification and the resistance–capacitance network modeling for the aerogel-insulated walls demonstrate good accuracy and reliability for subsequent analysis.

## Figures and Tables

**Table 1:** Thermophysical parameters of wall structure.

Material	Density kg·m^−3^	Thermal Conductivity W·m^−1^·K^−1^	Specific Heat kJ·kg^−1^·K^−1^
Internal plaster	1600	0.81	1.05
Foam concrete	900	0.22	1.05
GF (reference)	119.7	0.0380	1.290
AFC	266.2	0.0208	1.240
External plaster	1700	0.87	1.05

## Data Availability

Data are contained within the article.
